# Chambers’ Manual of Diet

**Published:** 1875-08

**Authors:** 


A Manual of Diet in Health and Disease. By Thomas King Chambers, M.D., Oxon., F.R.C.S.P., Lond., Hon. Physician to H. R. H. the Prince of Wales, Consulting Physician to St. Mary's and the Lock Hospitals, Lecturer on Medicine at St. Mary's School, etc. Philadelphia: Henry C. Lea; 1875. Octavo; cloth; pp. 510. Price, $2.75.

As a sequel to Dr. Chambers' admirable essays upon the Indigestions, the Renewal of Life and Restorative Medicine, he now presents us with a monograph upon Diet in Health and Disease. Most happily did our author strike the keynote of modern medical thought in his Restorative Medicine and with great appropriateness does he now give his sound and conservative views upon diet as the great fundamental means of the restoration. The work is designed to be eminently practical in its character, and hence lengthy chemical and botanical disquisitions upon the various dietory materials are excluded.

The volume is divided into three parts: 1st. General Dietetics, including theories of dietetics, choice of food, preparation of food, digestion, and nutrition; 2d. Special Dietetics of Health, including regimen of infancy and motherhood, childhood and youth, commercial life, literary and professional life, noxious trades, athletic training, hints for healthy travelers, effects of climate, starvation, poverty and fasting, decline of life, and alcohol; 3d. Dietetics of Sickness, including regimen of acute fevers, of other inflammatory states, weak digestion, gout and rheumatism, gravel, stone, albuminaria and diabetes, deficient evacuation, nerve disorders, scrofula, rickets and consumption, disease of heart, and arteries.

